# Balance of pro- and anti-inflammatory cytokines in livers of high fat diet rats exposed to fractionated gamma irradiation

**DOI:** 10.1186/s13104-018-3851-2

**Published:** 2018-10-19

**Authors:** Ayman Khalil, Hasan Omran, Fatima Alsheikh

**Affiliations:** Department of Radiation Medicine, Human Nutrition Laboratory, Atomic Energy Commission of Syria (AECS), P.O. Box: 6091, Damascus, Syrian Arab Republic

**Keywords:** Fractionated whole body gamma irradiation (FWBGI), HFD, Inflammation, Liver, IL6/IL10 ratio

## Abstract

**Objective:**

In this work, the effects of irradiation and high fat diet (HFD) intake have been examined in Wistar rat livers. HFD Wistar rats were exposed three times per week for 2 months to three different doses (0.5, 1, and 2 Gy) of a fractionated whole body gamma irradiation (FWBGI). Hepatic mRNA of these rats was evaluated for five cytokines, TNFα, IL1β, IL6, CRP and IL10. In addition, some critical protein levels were evaluated.

**Results:**

Results demonstrated that FWBGI was able to omit the inflammatory state already induced by the HFD through the depression of all pro-inflammatory genes. In addition, TNFα/IL10 IL1β/IL10, IL6/IL10 and CRP/IL10 ratios were less than 1 at all studied irradiation doses. IL6/IL10 ratio (mRNA and protein) was the best that represented an anti-inflammatory state with all used doses. Results could be of great importance in liver radiotherapy in HFD animal models and may give indicators about the inflammatory state improvement during FWBGI.

## Introduction

Nowadays, obesity and related diseases such as type 2 diabetes, hyperlipidemia and cardiovascular diseases are growing worldwide [1–3]. On the other hand, obesity itself is well known as a low-grade inflammatory state [4]. Inflammatory response to a high fat diet (HFD) is characterized by the activation of NF-κB and production of pro-inflammatory cytokines, such as TNFα, IL1β and IL6 [5, 6]. In fact, the inflammatory position can be introduced through a variety of mechanisms, for instance exposing an organism to biological, chemical or physical stimuli. Irradiation is considered a physical agent, which results in an inflammatory state and exposure can happen from different sources from our surrounding environment such as medical devices, nuclear accidents or other source as well as from therapeutic procedures.

Radiation-induced inflammatory response is characterized by the recruitment of Neutrophils and monocytes after tissue trauma, may also be the production of many pro-inflammatory and profibrosing mediators including IL1β, TNFα, IL6, CAXL1, or TGFβ [7–10]. Radiation-induced inflammatory responses are well described in the literature; however, the inflammatory response to HFD rats exposed to a whole or localized body irradiation is not well described.

Since liver is considered the primary defense target of our body [11], the current work aims to investigate the influence of fractionated whole body gamma irradiation on mRNA levels of several pro-inflammatory cytokines in livers of HFD Wistar rats, to analyze the ratio pro-/anti-inflammatory (mRNA and protein) cytokines and to evaluate the inflammation degree after exposure to a whole body gamma irradiation.

## Main text

### Animals

Five-week adult male Wistar rats weighting ~ 192 g obtained from (Charles Rivers Laboratories, France) were maintained under 12 h light/dark cycle having free access to food and water. Normal and HFD chow diets were (energy content 12% fat, 26% protein and 62% carbohydrates) and (energy content 74% fat, 26% protein, < 1 carbohydrates) respectively. Rats were housed in stainless steel cages at ~ 50–60% relative humidity and 22 °C.

At irradiation, rats were divided randomly into four groups (n = 9 for each group), HFD control (non-irradiated) and the other three groups were exposed to fractionated whole body irradiation of (0.5, 1, and 2 Gy), 3 times per week for 2 months. The correspondent cumulative doses of 24 fractions were 12, 24 and 48 Gy.

### Irradiation schedules

Fractionated whole-body gamma irradiation at a low dose rate of 100 mGy/m was administered to rats restrained in special irradiation-cages containing 9 rats for each dose using gamma ray apparatus (Theratron 80 Canadian design machine, Co-60, focal distance of 100 cm). Housing, standard food, drinking water, and HFD were unchanged before and after irradiation. Animals were closely observed for unwanted effects and there was no visible sign of discomfort or illness. At the end of the irradiation treatment, rats were euthanized using ether [12], sacrificed and their livers of each irradiation dose were collected and stored at − 80 °C until total RNA was extracted.

### RNA extraction and real time PCR

Total RNA was isolated from tissues using Roti-phenol reagent (phenol\chloroform\isomyl\alcohol: 25\24\1, ROTH^®^, Germany) and RNA was quantified (NanoDrop technologies, Qiagen, Germany).

The expression of target genes was determined using the Mx3oo5P QPCR systems (Agilent technologies, Germany). Total RNA (1 µg) was amplified using 2× PCR SyGreenone-step low Rox kit (ROTH^®^, Germany). PCR was performed with 0.5 µM of each primer and the cycling conditions were as described [13]. Rat TNFα, IL1β, IL6, CRP, and IL10 expressions were normalized to β2-actin expression and data quantified by the method of 2^−ΔΔCt^ [14]. The primers used are:

- β2-actin-F: 5′-AAGGCCAACCGTGAAAAGAT-3′.
- β2-actin-R: 5′-TGGTACGACCAGAGGCATAC-3′.
- TNFα-F: 5′-GGGACAGTGACCTGGACTGT-3′.
- TNFα-R: 5′-TTCGGAAAGCCCATTTGAGT-3′.
- IL1β-F: 5′-TCGCTCAGGGTCACAAGAAA-3′.
- IL1β-R: 5′-CATCAGAGGCAAGGAGGAAAA-3′.
- IL6-F: 5′-TCTATACCACTTCACAAGTCGGA-3′.
- IL6-R: 5′-GAATTGCCATTGCACAACTCTTT-3′.
- CRP-F: 5′-TTCCCAAGGAGTCAGATACTTCC-3′.
- CRP-R: 5′-TCAGAGCAGTGTAGAAATGGAGA-3′.
- IL10-F: 5′-CACAAAGCAGCCTTGCAGAA-3′.
- IL10-R: 5′-AGAGCAGGCAGGATAGCAGTG-3′.

Division pro-inflammatory cytokine values by the IL10 value calculated TNFα/IL10, IL6/IL10, CRP/IL10 and IL1β/IL10 ratios.

### IL6 and IL10 protein level measurements

Interleukin 6 (IL6) and Interleukin 10 (IL10) ELISA kits (Sigma Aldrich^®^, St. Louis, MO 63103 USA) were used to determine IL6 and IL10 protein levels in control and irradiated rat livers according to the manufacturer’s instructions.

### Histological examination of liver tissue

Liver specimens were embedded in cryostat embedding medium (Killik, Bio-Optica, Italy) and frozen at − 80 °C. Specimens were cut into 5 µm thick sections by cryostat (SLEE MNT Cryostat Medical GmbH, Germany) that were then stained with Hematoxylin (Fluka chemie Gm bHcH, Switzerland) and Eosine (Qualikems laboratory Reagent, India) according to standard procedures. Images were taken using a light microscope (BX53, Olympus, Japan) linked to a personal computer.

### Statistical analysis

Statistical analysis was performed using Mann–Whitney test by means of Origin 8 Software. Values were considered statistically different when p ≤ 0.05 and results are presented as mean ± SD.

## Results and discussion

**Fig. 1:**
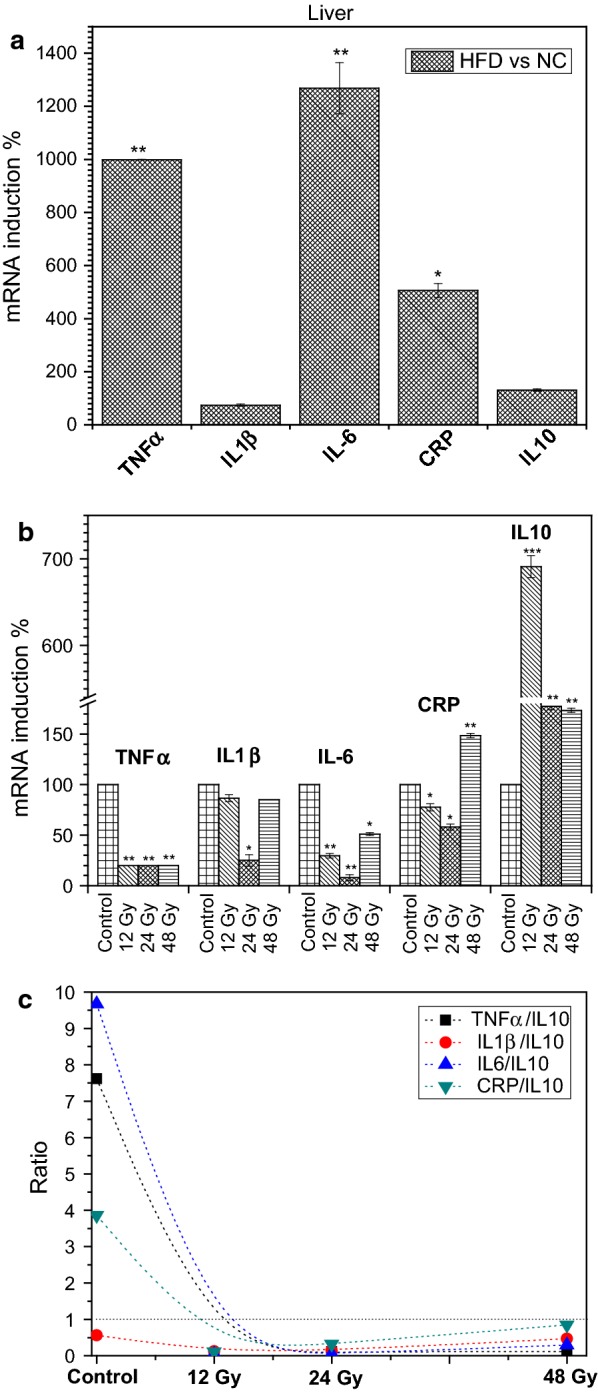
**a** Effect of high-fat diet (HFD) versus normal chow diet mice (NC), **b** effect of fractionated whole body irradiation (FWBGI) of high-fat diet (HFD) versus control HFD, on relative gene expression levels in the livers of 6-week-old male Wistar rats, **c** ratios values of pro-/anti-inflammatory mRNA gene expressions. Doses 0.5, 1 and 2 Gy equal 12, 24 and 48 Gy of cumulative doses of FWBGI, *p < 0.05, **p < 0.01, ***p < 0.001

First, HFD is well documented to induce an inflammatory state in animal models [15–17] and similarly our diet was capable of inducing inflammatory state in treated Wistar rats. As demonstrated in Fig. 1a. Expressions of some pro-inflammatory genes were up regulated by HFD such as TNFα, IL6 and CRP, the induction of theses cytokines were respectfully, 1000-, 1300-, and 500.10% (p ≤ 0.01). Whereas, the anti-inflammatory response by IL10 did not significantly changed. These results were in agreement with other studies, which demonstrated that HFD can induce in the liver an inflammatory response mediated mainly by TNFα, IL1β, IL6 and CRP.

Results from Fig. 1b indicate that 12, 24 and 48 Gy of FWBGI down regulated the expression of the pro-inflammatory cytokine TNFα already induced by HFD to nearly (20%, p ≤ 0.05) with all used gamma doses. While the expression of IL1β was not significantly altered except with the 24 Gy dose where, its mRNA level was apparently down regulated to 30% (p ≤ 0.05) as demonstrated in Fig. 1b. The expression of IL6, another pro-inflammatory cytokine that plays an important role in the inflammatory state regulation, was significantly reduced to ~ 30 and 50% by 12 Gy and 48 Gy doses, respectively. Interestingly Fig. 1b indicated that 24 Gy of gamma irradiation reduced the expression of IL6 to about 10% (p ≤ 0.05). C-Reactive protein (CRP) another pro-inflammatory marker that is produced in the liver was down regulated by 12 and 24 Gy doses but at 48 Gy exposure; its expression was significantly increased by about 150% (p ≤ 0.01).

In fact, radiation-induced liver inflammation is well known, a study showed that a single dose of gamma irradiation was able to induce pro-inflammatory cytokines such as IL1β and IL6 and many chemokines in the liver of Wistar rats [18]. In addition, previous studies have established that irradiation induces an increase in the main pro-inflammatory chemokines via the induction of local pro-inflammatory cytokines [19–21]. Indeed, the current results are in disagreement with these previous findings, which demonstrated the post irradiation inflammatory state. Our results on the liver of HFD rats confirmed that irradiation may delete the effect of HFD-induced pro-inflammatory cytokine via the depression of mRNA expression of studied cytokines. In addition, our experiments indicated clearly that the inflammatory state is obliterated by the effect of all used doses. This omission of such inflammatory response in our work was via the overexpression of an anti-inflammatory response mediated by IL10 (Fig. 1c).

In fact, these results, which support the anti-inflammatory effects of irradiation, may be explicated by the dose response of specific tissue and by the fact, that cytokine production is a time-dependent [22, 23]. Moreover, hyper fractionated irradiation may result in activated macrophages and accumulation of IL10 [24].

Anyhow, to assess the inflammatory state with studied doses, we analyzed the expression of IL10 and pro-/anti-inflammatory ratios. Interestingly results from Fig. 1c indicated that gamma irradiation increased the expression of this major anti-inflammatory cytokine by ~ 700-(p ≤ 0.001), 180 (p ≤ 0.01) and 170% (p ≤ 0.01) at 12, 24 and 48 Gy doses respectfully. While, pro-/anti-inflammatory ratios were TNFα/IL10 = (0.03, 0.1, 0.1) at 12 Gy, 24 Gy and 48 Gy respectfully. With the same order of doses, the ratios of IL6/IL10 (0.04, 0.04, 0.3), and ratios CRP/IL10 were (0.1, 0.3, 0.9). From these ratios, we can conclude that the inflammatory state is not present in all used doses of gamma irradiation compared with ratios resulted from the effect of HFD in which, the ratio calculations indicated an inflammatory condition as revealed in Fig. 1c except for IL1β. Interestingly, with all studied doses, all ratios paragraphs tend very nearly towards the same form with an increasing exposure of gamma irradiation.

Therefore, all ratio values for TNFα, IL6, IL1β, and CRP/IL10 were < 1. These results clearly indicate that all studied doses reduced the inflammatory conditions already induced by the effect of HFD in the liver of Wistar rats, and the ratio IL6/IL10 is the best that represents the anti-inflammatory conditions.

Actually, ratios have been used as biomarkers in several previous studies. TNFα/IL10 and CRP/IL10 were inflammatory biomarkers of coronary artery disease in Indian patients [25]. In addition, the ratio TNFα/IL10 was successfully used to assess the risk of an in vitro fertilization failure [26]. Another recent study indicated that this ratio is related to burn severity, and may be used as a biomarker of the risk prediction susceptibility [27].

**Fig. 2:**
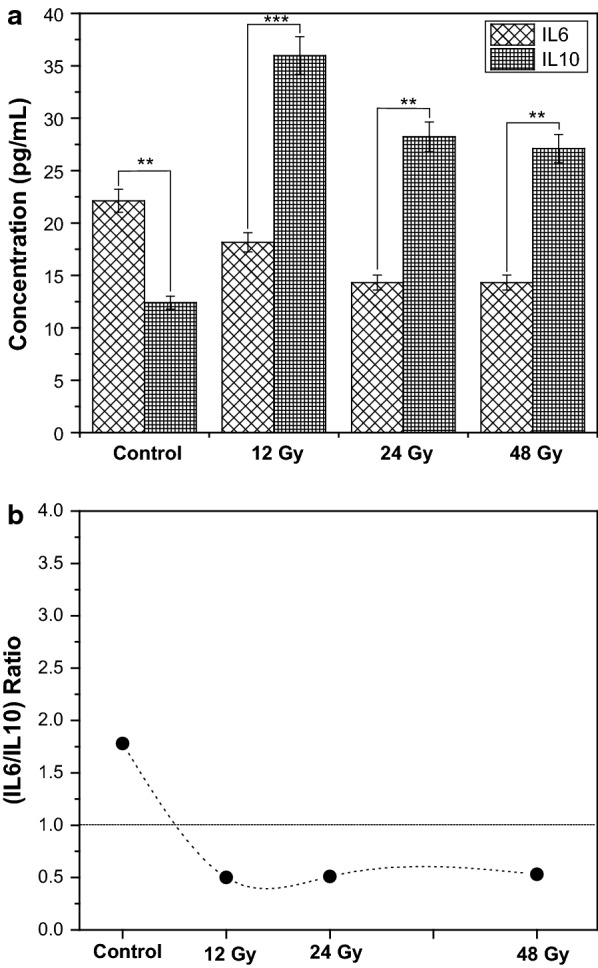
Effect of fractionated whole body irradiation (FWBGI) of 12, 24 and 48 Gy on protein levels of pro-inflammatory cytokine IL-6 and anti-inflammatory one IL10 in the livers of high-fat diet (HFD) male Wistar rats versus control (HFD); **b** ratio IL6/IL10 for control and at each FWBGI doses calculated from **a**

To enforce our results obtained from mRNA, the amount of protein levels of the most significant ratio IL6/IL10 was analyzed. Figure 2a and b clearly indicated that there was a significant decrease by the average of 36% (p ≤ 0.05) with all used doses in IL6 protein level. Controversy, a significant increase of IL10 by about 220% (p ≤ 0.05) on average (Fig. 2a) was documented. These results were supported by the IL6/IL10 ratio calculations. Clearly, Fig. 2 showed that the ratio was decreased from about 1.8 in the control to about 0.5 post FWBGI with all used doses (Fig. 2b).

**Fig. 3:**
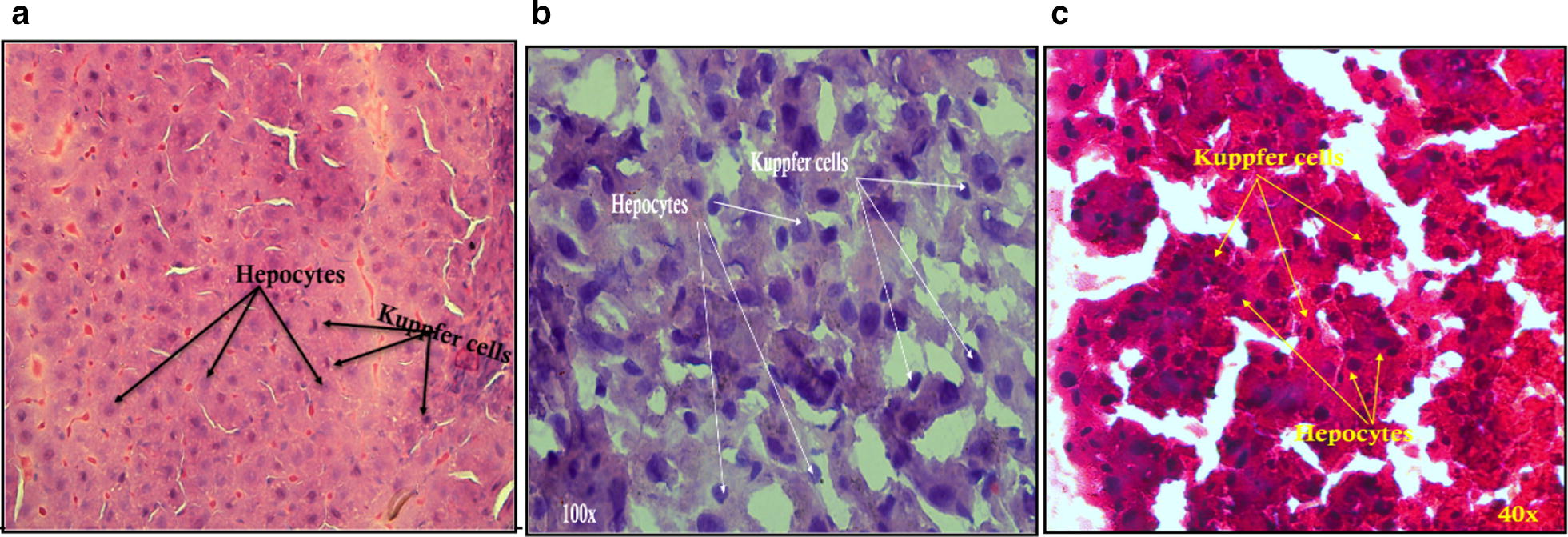
Hepatocytes and Kuppfer cells (KC) in liver histology of male Wistar rats (**a**) liver of normal chow diet rats (**b**) liver of HFD rats (**c**) HFD rats livers irradiated with 1 Gy (24 Gy) of fractionated whole body gamma irradiation(FWBGI)

Figure 3 represents two types of liver cells with various diet, hepatocytes and kupffer cells (KC), we can clearly remark increased in the KC in HFD and irradiated HFD rat livers (Fig. 3b, c). KC are the first line of defense against various pathogens entering the liver through the portal vein, and are known as the resident liver macrophages. These cells play an important role in several mechanisms such as cytokines secretion, metabolism and hemostasis [28]. In our histological chows, KC increased in irradiated Wistar rat livers. Figure 3c indicates a remarkable increase in KC as a result of the irradiation exposure (i.e. 24 Gy) revealed that these cells may keep the liver from irradiation induced-injury [29]. Moreover, this outstanding number of KC may protect the liver against radiation induced cytokine production [30] maybe producing an anti-inflammatory response (i.e. IL10) as mentioned earlier.

Actually, histological study may confirm our current work indicating that a low dose rate has a protective role in the liver of irradiated HFD Wistar rats by producing an anti-inflammatory response, which KC may play a certain roles, but the mechanism by which this response occurs still requires further investigations.

In conclusion, this work gives an elementary indication about the effect of dietary nutritional behavior. Results demonstrate that a diet rich in fats remodeled the expression of the inflammatory state during FWBGI in the liver of Wistar rats. In fact, the balance of pro-/anti-inflammatory cytokines (mRNA and protein) was an indicator of the effects of irradiation exposure in HFD Wistar rats. Ratio values of TNFα/, IL1β, IL6 and CRP/IL10 were present as anti-inflammatory state with all studied doses of gamma irradiation, and were minor compared to the same ratios with normal chow. Furthermore, these findings indicate a favorable effect of fractionated whole body exposure on pro- and anti-inflammatory balance in the liver of HFD Wistar rats.

## Limitations

FWBGI is a complex process that initiates several other molecules cascades related to inflammatory process including many other cytokines and cytokine receptors, which are important to furthermore study, particularly those molecules that have radio therapeutic action. However, the mechanism underlines the enhancement of anti-inflammatory pathway mediated by IL10 in this animal model by FWBGI still requires additional and deeper investigation to find out which signaling pathways involved in such effects.

## Abbreviations

HFD: high fat diet
TNFα: tumor necrosis factor alpha
IL1β: interleukin 1 beta
IL6: insulin receptor substrate 4
CRP: C-reactive protein
IL10: interleukin 10
KC: Kuppfer cells
FWBGI: fractionated whole body gamma irradiation
mRNA: messenger RNA
PCR: polymerase chain reaction
